# On Robust Methodologies for Managing Public Health Care Systems

**DOI:** 10.3390/ijerph110101106

**Published:** 2014-01-17

**Authors:** Shastri L. Nimmagadda, Heinz V. Dreher

**Affiliations:** School of Information Systems, CBS, Curtin University, Perth, 6102 WA, Australia; E-Mail: shastri.nimmagadda2011@gmail.com

**Keywords:** diabetes, food ontologies, data warehousing, data mining, visualization, data interpretation

## Abstract

Authors focus on ontology-based multidimensional data warehousing and mining methodologies, addressing various issues on organizing, reporting and documenting diabetic cases and their associated ailments, including causalities. Map and other diagnostic data views, depicting *similarity* and *comparison* of attributes, extracted from warehouses, are used for understanding the ailments, based on *gender*, *age*, *geography*, *food-habits* and other *hereditary* event attributes. In addition to rigor on data mining and visualization, an added focus is on values of interpretation of data views, from processed full-bodied diagnosis, subsequent prescription and appropriate medications. The proposed methodology, is a robust back-end application, for web-based *patient-doctor* consultations and e-Health care management systems through which, billions of dollars spent on medical services, can be saved, in addition to improving quality of life and average life span of a person. Government health departments and agencies, private and government medical practitioners including social welfare organizations are typical users of these systems.

## 1. Introduction

In recent years, one of the chronic diseases, alarming among mass population worldwide, is diabetes and it is increasingly widespread irrespective of geographic region, race and/or age. Many healthcare institutions in many countries spend billions of dollars, although the quality of services to diabetic patients has improved in many rich countries. A renewed campaign has emphasized preventive care for diabetic health, especially on documenting, revitalizing and bringing awareness about healthy food habits to the massed population of diabetic patients worldwide. One of the main reasons are unhealthy life styles and food habits, which affect blood sugar levels, cholesterol and blood pressure. One can imagine that because of unhealthy life styles, there are series of chain-linked chronic diseases. Authors have put forth attempts to design, develop and implement information technology/information system solutions for the purpose of documenting the cases, understanding the chronic disease and its connectivity to unhealthy life styles. Recently, several contributions are made in documenting these cases and analyzing historical data for future forecasts [1,2,3]. In this context the Authors have developed ontology-based multidimensional data warehousing and mining for organizing huge amounts of historical data [4]. 

**Figure 1:**
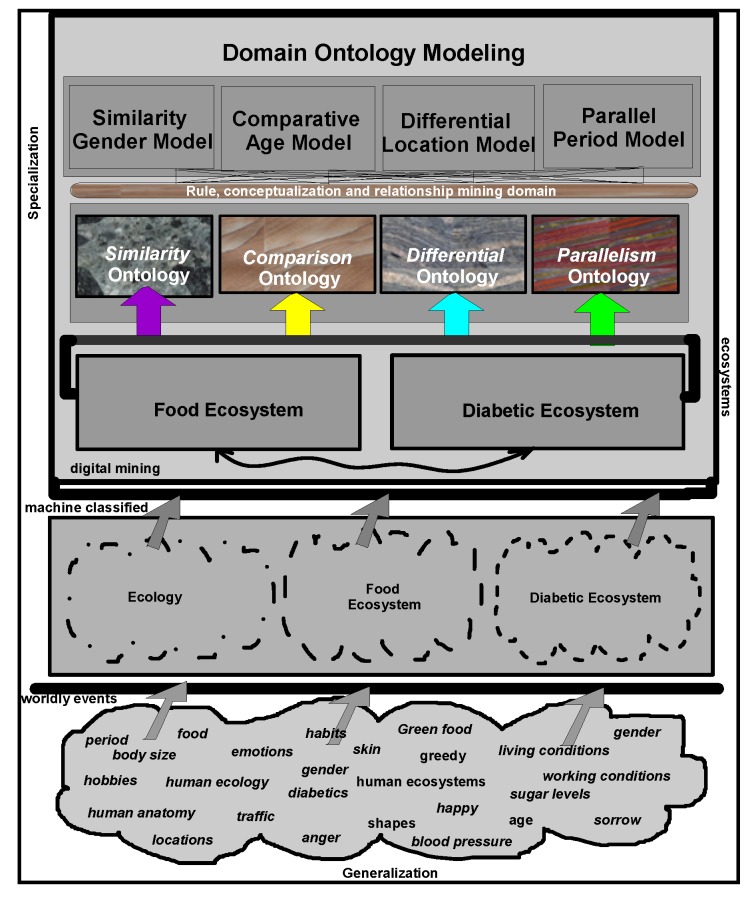
Connecting multiple domain ontologies.

Even though multiple domains-research is ongoing, an integrated research effort on the connectivity of *food-diabetic* domains is lacking, especially using the existing information systems (IS) and the proposed IS solutions. Worldwide, governments spend billions of dollars on preventive health-care systems and high priority medications. Countrywise diabetic cases and associated diseases, have so far been difficult to document and or report, because of complications in organizing these data and information on geographic and periodic scales. Social welfare organizations, medical institutions, clinical specialists, nutrition professionals and dieticians often encounter problems of data and information access on both these scales. Awareness includes early preventive measures and already high-risk patients on high priority treatments, such as medication and implementing healthy food habits. Food intake affects blood sugar, which is root cause of the diabetic disease. In developed countries, this is a common disease, though this trend is changing in recent years. In developing countries such as, India, China and other Latin American countries, there is an increasing attention and awareness. In a mass population, initiatives must include electronic consultations, electronic prescriptions, greater reliance on evidence-based medication, care collaboration centers with easy access to diabetic patients’ medical records and improved inventory management. These initiatives may complicate the design, development and implementation processes on a massive scale. For this purpose, ontology-based multidimensional data warehousing and mining are proposed for physical and logical organization of heterogeneous patients’ and medical professional’s data. As shown in Figure 1, different domain ontologies are modelled. The methodologies ensure that multidimensional data located in a centralized pool guarantee the cost savings and quality care programs at local and global-centers.

Ontologies are needed to address the semantic and schematic ambiguities, discrepancies that usually occur during naming conventions, vocabularies, conceptualization and contextualization of attributes of various dimensions used and/or emerged, during conceptualization process of data modelling. Different domains, such as food and diabetes are described and their connectivity is intended to be established among common attributes of dimensions of different domains. If the attributes narrated through conceptualization and contextualization among attributes of associative domains have an ambiguity in describing their naming conventions and vocabularies including schemas, they can be resolved by semantic and schematic ontologies. Even the data structures, while connecting between domains may have created problems, but are set to have these resolved through description of semantic and schematic based ontologies. For example, the attributes described in *diabetes* and *food* domains, if their attribute strengths are exchanged in either domain, they are still positively correlatable. 

In order to handle multiple domains, heterogeneous and multidimensional data, a robust methodology is proposed to address the issues of documentation and organization of data, including public awareness through education. This paper proposes an intelligent information management system that can store and integrate different domain ontologies, such as *diabetes* and *cholesterol, food* and *anti-oxidants,* in multidimensional schemas. The systems can create a prescription or meal plan according to a person’s lifestyle and particular health needs. The required knowledge is stored in an ontologically structured metadata model, predefined by domain experts. It contains diabetic ontology, including embedded set of *personal* and *food-domain* ontologies. The prescriptive medication of diabetes, is intelligently and effectively managed by ontology-based data mining, data visualization and interpretation. Medical professionals use these data views for interpretation of seriousness of ailments, their symptoms and appropriate prescriptions. 

An information system using a healthcare ontology provides a standardized representation of healthcare data documentation. One embodiment of the information system comprises a digital logic platform storing and using the healthcare ontology. The healthcare ontology describes concepts and relationships among the concepts and contexts, derived from the corpus of domain-specific knowledge and linking it with standardized terminological systems. Ontologies target modelling worldly events (Figure 1) and construct expressions of complex classes, including narrating data characteristics with and without ambiguities. Ontologies are used in applications that need focus of semantics, schematic and syntactic heterogeneities. Ontology describes the meaning of the content that articulated among data attributes, used in the current data modelling processes. 

## 2. Issues, Challenges and Problem Statement

**Figure 2:**
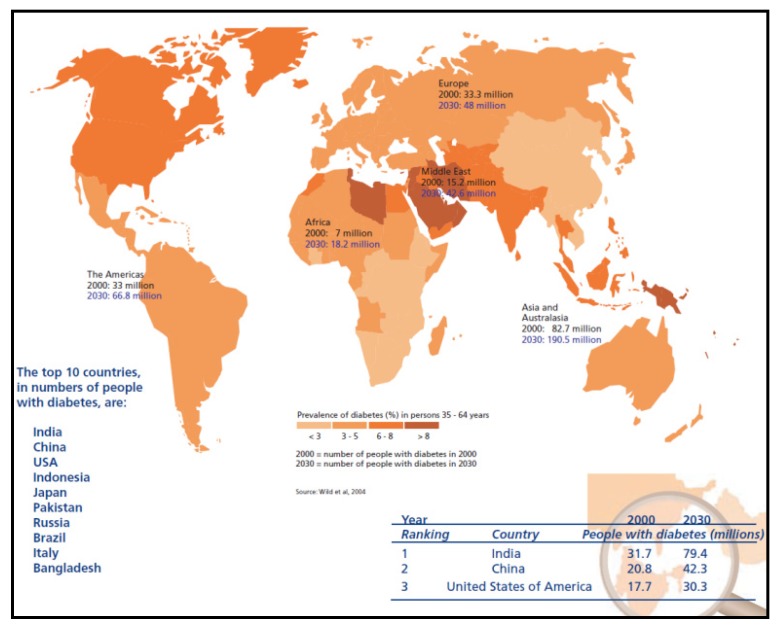
Alarming diabetes spread worldwide (Source: World Health Organization, WHO).

This paper is intended to study the feasibility and applicability of robust database methodologies such as, an ontology-based multidimensional data warehousing and data mining for integrating multiple domains, such as diabetes and foodstuffs (and nutrients) that include antioxidants. This can evaluate the effectiveness of diabetic disease management, using both patient medical records and medicare claims that address clinical outcomes, costs and process measures. If the interventions can positively influence various measures taken, the project may been expanded to an advanced care stage. Reduction in medicare payments suggest inpatient hospital services and emergency room visits, as well as increase in the percentage of patients, being tested annually for HbAlc and being referred for diabetic education. More importantly, HbAlc test results are expected to improve understaning of this ailment, if any. It is a primary measure of consistent blood glucose control. As per WHO statistics (Figure 2), the alarming situation is that people in India, China and USA are suffering from diabetes in numbers respectively 32, 21 and 18 million, and these figures are expected to reach 79, 42 and 30 million by 2030. Other countries with millions of sufferers include Australia, Indonesia, Japan, Pakistan, Russia, Brazil, Italy and Bangladesh, which suggest the availability of enormous data sources worldwide.

The comprehensive education programs may include resolving various issues, such as educating the mass population, awareness and the consequences of this deadly disease, and including healthy lifestyles and food habits. An educational program that refers to professionals involved in the management of public health care system is included in the Table 1. In addition, preventive care helps controlling the disease, especially of hereditary nature. Regular doctor check ups, healthy meal plans and regular body exercise, maintaining and controlling the existing glucose levels, in essence, are to educate mass population worldwide.

**Table 1:** Comprehensive Educational Campaign.

Health History	Current Self-Care Management Practices
Previous use of Medication	Lifestyle practices
Diet/Meal planning history Current mental health	Physical and psycho Social Factors
Family and social supports	Factors affecting learning
Previous diabetic education, actual knowledge and skills	Family Histories and any other family histories

As narrated in Table 1, these programs may be organized in the countries, where severe cases are reported on a priority basis. As per statistics narrated in [5], the global health expenditure on diabetics reached in total USD 376 billion in 2010 and 490 billion USD is expected to be spent in 2030. Globally, 12 percent of total expenditure is spent on diabetes, USD 1,330 per person in 2010. Expenditure is variable, based on geographic region, age, gender and countries’ GDP attributes. Because of diabetic-related ailments, the losses in national incomes are enormous, for example, for years 2005–2015, the losses are estimated to reach USD 558 billion in China, USD 303 billion in Russia, and USD 237 billion in India. Surprisingly, for USA, in spite of an alarming diabetes situation in all age groups, because of the billions of dollars spent, diabetes and their associated ailments have been curtailed. Unfortunately, poorly populated countries are the real victims of diabetic-related diseases, because of very low expenditures per capita, suggesting urgent basic diabetes care needs. Even in rich countries, in North America and European countries, disadvantaged indigenous, ethnic minorities, recent migrants and slum dwellers suffer high rates of diabetes and its complications. The major challenge is to reduce social inequalities among and within countries that restrict opportunities for good health and access to healthcare. Economic implications and considerations on issues, associated with worldwide diabetes expenditure disparities, are beyond the scope of the current research. 

In developing countries, connectivity, communication and interaction among patients, medical professionals, dieticians, druggists and social welfare organizations involved in diabetes control, are critical issues. Projects associated with preventive care, drug research, inadequate access to medical records and operational research are other challenges. There could be several preventable and avoidable medical complications; patients may die because of lack of awareness and unhealthy lifestyles. There is no proper record and documentation of these patients. The costs incurred due to serious avoidable errors are enormous. Poor data quality leads to practice variation, medical errors, out-of-control emergency room visits, and fraudulent claims. Providers increase prices, payers increase premiums, and patients lack quality access care. Besides these issues, data entry is prone to error, especially during claim filing, procedure coding and medical records. When there are changes in technology and disparate systems, the chance of introducing errors increases. In addition, machine and equipment maintenance and their responses are significant and these data add value to the domain modelling process and to the ultimate metadata structure and its interpretation. Data integration through warehouse modeling, mining and visualization can facilitate an accurate interpretation that involves saving human lives. Public awareness is another issue. Awareness among the mass population leads to appropriate remedies for diabetes and healthy lifestyles. Diabetes education occurs in a variety of settings, depending upon needs of the patient and severity of the disease. Inpatient, outpatient, home health and pharmacy settings, all provide avenues for effective individual and group education. 

Proposed data modeling and warehousing concepts support the description and integration of multiple data sources within and outside the organizations. Repetitive or incompatible data from these systems can be a significant source of data quality problems. In addition, consistently standardized data formats and datasets for transactions, are crucial for maintaining good quality data and thus for improved health care system designs. Often the data captured by health clinics are noisy, contain errors and inconsistencies. Many patient records may have typographical errors, missing values or incorrect information regarding the patients. Many records are duplicated. Data cleaning takes enormous amount of time and many records collected are not in compatible formats for ontology descriptions and modeling, processing data in a warehouse environment and or for interpretation is time consuming. 

Another issue is generation of too many mining rules from the data that can confuse interpretation by medical practiners and even patients. Doctors are too busy with patients; they cannot afford time to sieve large number of mining rules generated by the databases. It is important to present the data in an easy understandable way, interpretable by the medical practitioners. Data trends, correlations and patterns are so critical in interpretation of classifications of patient and medical records. Special skills are needed to improve and understand the data mining, data visualization and interpretation tasks. Data warehouse approach reconciles data organization that include format differences and encoding schemes during integration, including resolving semantic, schematic and syntactic heterogeneities. A user-oriented approach illustrates exploration and discovery of data patterns. Data visualization is used to enhance an understanding of the pictorial representation of diabetic records and their clinical interpretations. Proposed methodologies are detailed in the following sections. 

## 3. Motivation and Need of Ontologies

Our intention is to design and describe an integrated framework work, which can effectively lessen the research effort of managing medications of diabetic patients. Keeping in view the variety of users, doctors, dieticians and professionals involved in preventive care, a systematic approach involving ontology-based data integration is needed. Archetype patterns [6] used with high level ontology abstraction, at global scale are analyzed, keeping in view bio-informatics and health-care domains on a geographic scale [7]. 

**Figure 3:**
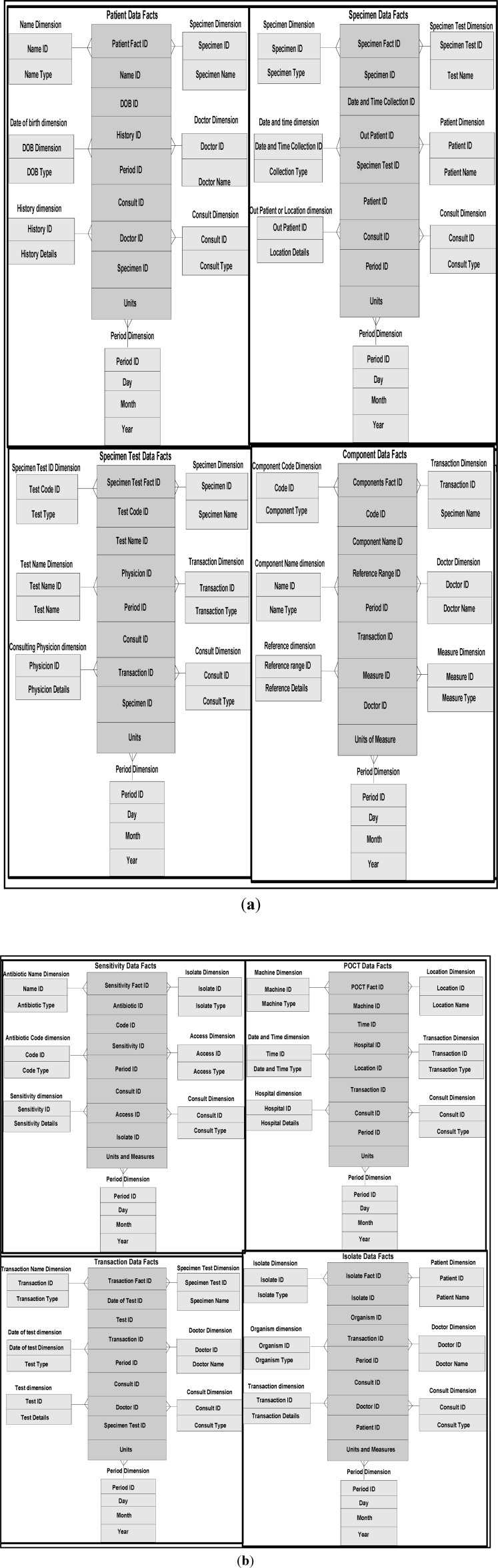
(**a**) Heterogeneous data and their connectivity, modelling patients and specimen dimensions that narrate clinical histories of diabetics disease. (**b**) Sensitivity and other transactional multidimensional data modelling that narrate clinical histories of diabetics.

**Figure 4:**
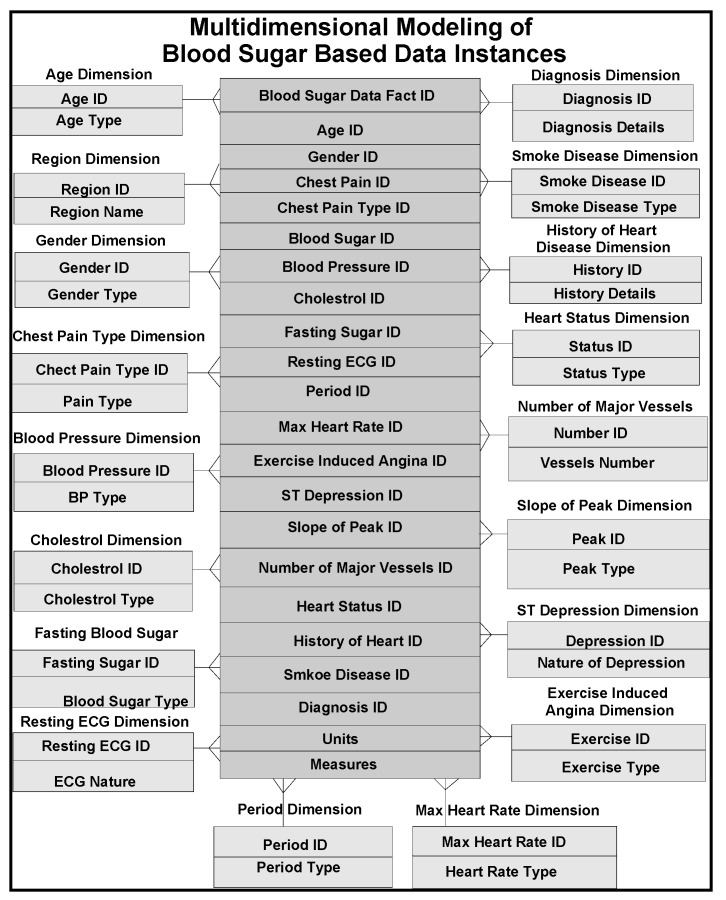
Multidimensional modeling of blood sugar data instances.

**Figure 5:**
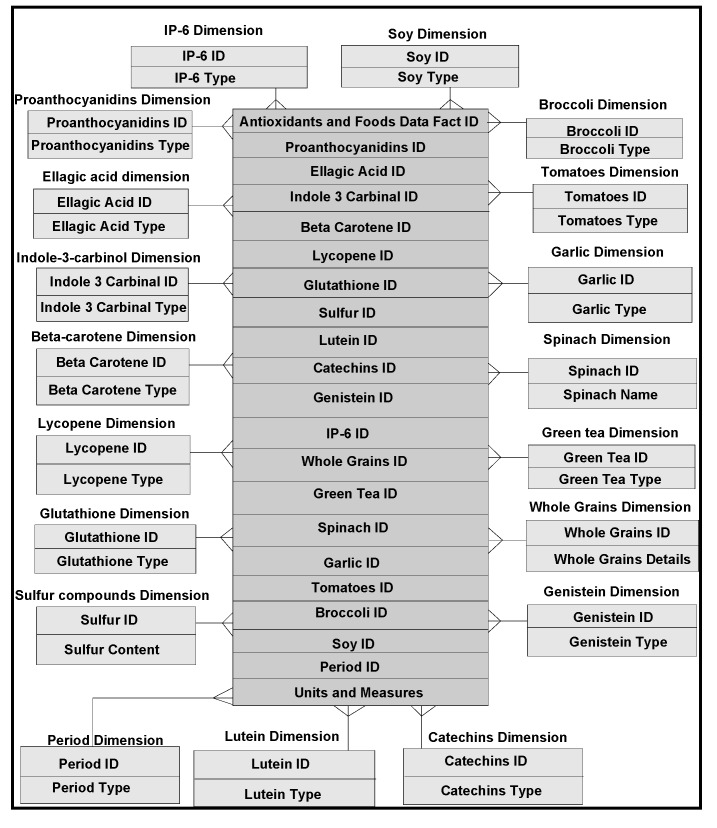
Multidimensional modeling of data instances relevant to antioxidants and their associated foods affecting the blood sugars.

In our frameworks, ontology is a description of concepts/contexts and relationships among various either entities and or dimensions that exist in the knowledge domains of *food-diabetes*. Ontology deals with queries on what entities and or dimensions exist in a given domain and how such entities or dimensions can be grouped, related within hierarchical, relational and networked data structures. Ontology Web Language (OWL) is a knowledge representation, and in our case, knowledge is expressed in the form of various fine-grained data schemas as represented in Figure 3, Figure 4 and Figure 5, which explore connections from multiple domains, which can make data mining effective, including visualization and interpretation. Multidimensional data relationship diagrams drawn, are modelled in Oracle-driven, Windows-based high speed computing workstations. Availability of heterogeneity and multidimensionality nature of data sources on *food-diabetes* has, in fact, motivated us the necessicity of developing an ontology-based warehouse for accommodating multidimensional healthcare metadata structures. 

## 4. Methodology

### 4.1. Ontology-based Warehouse Modelling of Multidimensional Diabetes Data Instances

The proposed methodologies are comprehensively used in various business environments [8,9,10]. They are further extended in other domains robustly, such as the human ecosystems and human anatomy domains [4,11] to test their validity and versatility. For this purpose, ontological descriptions written in the form of schemas in different domains, are integrated in a warehouse environment to make connectivity among domains. A metadata is constructed from which several data views are extracted for visualization and interpretation purposes.

*Similarity* and *comparison* ontologies (Figure 1) are worth analyzing in understanding of *similarity* of patients’ ailments, in adition to comparing different domain data of similar and dissimilar data instances of patients, symptoms, food habits, medications and preventive care regimes. For example, same gender persons may have similar food habits, with similar symptoms and or among *age* attribute groups. They may need similar medications/prescriptions and preventive care. Another example is, preventive care of early symptoms for those patients, not yet diabetic nature (with symptoms persisting); medication and treatment phenomena may be in high risk and priority patients, in which, these ailments reported, are under medication for long periods of time. Another example is, in such areas (geographically distributed, such as Africa, Asia, and Latin America), where people have similar lifestyles, with similar ailments and similar or dissimilar food-habits. Similar age groups (such as 45–55 age groups) have similar diabetic type symptoms and or prescriptions. Adults may have diabetes type 1, with similar age and food-habit groups or categories. Event Similarity Prediction (ESP) is an algorithm that can compare, compute attributes and narrate histories of ailments among mass populations. Frequent occurrence of diabetic symptoms in a period of time under analysis is also significant. Other events are, blood sugar and pressure levels under definitive age and gender groups. Non-medical events could be daily intake of food and daily time spent on body exercises. All these data instances are populated in dimension and fact tables, with details of patients, doctors’ notes and facilities, where preventive care and medication are in progress. 

As described in [11], several models comprising of dimensions and their attributes in a warehouse environment, focus on rules and constraints, templates, mappings, domains, formats, measurement types, instruments, dimensions, attributes, fields, treatments, patients, medical practitioners, medical prescriptions, bench marks, test results, specimens, specimen types, specimen sizes, periods and units. In addition, glucose levels, glucose type, ophthalmology, eye tests, daily food intake, types of food, food hygiene, normal daily routines, and amount of time spent in daily exercises, cholesterol level are other relevant dimensions. These multidimensional schemas are integrated with other schemas, related to lab test data instances from several other dimensions and fact tables. Star schema models that can handle multidimensional data instances are represented in Figure 3. A few of such tables are described below with their characteristics in the subsequent sections.

*Patient Dimension* (*Pat Dim*): *Name*, *date of birth*, *history number* dimensions are highlights of this table. This table stores demographic information of all patients with laboratory tests. The data warehouse generates internal identifiers for all patients and responsible for linking all the other pertinent dimensions and fact tables, such as specimen test dimension and fact tables. The data warehouse considers the concatenation of *patient* *name*, *date of birth* and *history number* as primary keys used in the identification patient dimension fact tables. In a more conventional approach, several primary keys of other dimension and fact tables, including outpatient care systems of given patient with different patient’s history numbers of their dimension tables, are connected, including the correct spelling of patient’s name, units of history numbers and dates of birth. A variety of heuristics are used at query time to determine when it is safe to assume that different primary key instances are associated with a single patient. In most cases, data warehouse designers chase the records of the patients and check physically which records are kept or discarded in consultation with physician. 

*Specimen Dimension* (*Spc Dim*): This table organizes, storing all the specimen records which are collected from laboratory tests. This dimension table has typical attributes such as *identification number*, *the specimen collection date and time*, and *hospital or outpatient collection location*. Specimens are typically identified by the combination of specimen numbers and collection date and time. The data warehouse generates an internal identifier for all the patients’ specimens. This internal identifier is used in the Specimen Test Dimension Table to link specimens and lab tests fact data tables.

*Specimen Test Dimension* (*Spc Tst Dim*): *Test code or identification*, *test name*, and *consulting physician’s name*, are typical dimensions in this table that represent the specimen tests. Internal patient and specimen identifiers are included to link specimen tests of patients and specimens, respectively, linking dimension and fact tables. Fact tables consist of storage of all data instances along with units of the specimens. Tests are identified by the combination of specimen identifier, test code and ordering physician instances. The data warehouse generates an internal identifier for specimen test entries. This identifier is linked with the transaction dimension and fact tables.

*Transaction Dimension* (*Tran Dim*): The transaction dimension possesses attributes of *date and time* and *tests* with their corresponding data instances in fact tables. Transaction dimension provides a mechanism by which the progressions of lab tests are documented. In the fact table, for each transaction, there is an internal identifier created, by the warehouse. Provisional test results may be generated before the final results are made available, to the patient or consulting physician. The Transaction Dimension Identified is connected to the preliminary and final test result dimension tables. Since the entire lab tests are tagged with transaction identifiers, lab results can easily be sorted out by transaction identifiers, such as transaction fact table identification, which can reveal the progression of the lab results from preliminary to final data instances. 

*Component Dimension* (*Comp Dim*): The composite dimension and lab test results are stored in these dimension and fact tables. Typically they consist of *component code*, *component name*, *reference ranges,* *units of measure* and *results*. As narrated before, a transaction identification id, from the transaction dimension and fact tables is linked with all components to track the progression of component data instances.

The component dimension and fact tables are the finest level of granularity for a majority of lab tests (dimension tables). The typical result column contains the results of lab tests. There could be some other lab tests for which the value of the result column is just a reference (through attributes) into other dimension and fact tables. For this purpose, the component dimension table that contain a result type column, records the method of interpretation including diagnostic notes in the result column. Thus at places, the result column appears as the root of a hierarchical structure for specified lab tests. The component result is stored as reference data instances for lab result reports as blocks of test and microbiology culture results. Some lab results are reported as multiple lines of text. This is supported by creating a *text* dimension (and corresponding fact tables) identifier for the block of *text*, storing the identifier in the component result field and finally storing the block of text in the free *text* dimension table (fact tables). Similarly, microbiology culture data are stored in a separate dimension and fact tables. When culture results need to be reported, the transaction dimension identifier that is stored in the component result column is triggered and isolates (conceptualized/contextualized) relationships that are built and accordingly stored in an isolated table with the *transaction dimension ID*.

The component table is flexible enough to be used for other data types as well. To store other forms of complex lab results, a table is created with a new data type value to store the component result type column. When new data are made available, a unique identifier for the data is generated, the identifier is stored in the component result column and filled the component result type column appropriately. The lab results are added to the new table along with the data identifier that was added to the component result column. 

*Isolate Dimension* (*ISL*): Portions of culture results from tests performed on specimens, are stored in tables, associated with these dimensions. This table stores *the organism* or *organism class* that was identified, *the identifier for the isolate*, and *the transaction identifier* that is stored in the component result column. The isolate dimension and tables are used in conjunction with the sensitivity tables by linking them.

*Sensitivity Dimension* (*SEN, SCN, SCR*): The antibiotic sensitivity/MIC results for all isolates are stores in these dimension tables. Typically, the table contains *antibiotic name*, *antibiotic code*, *sensitivity results*, and *isolate identifiers*. The sensitivity table stores, unscreened results as well. Screened results are stored in the sensitivity table, developed for screened results. It has the same design as the original sensitivity table but with stricter access permissions.

*Free-Text Dimension*: Blocks of texts are stored for various data types, previously mentioned table dimensions. Every block of text, has an *identifier*, *text fact ID*. This identifier is referenced by other tables to link data in them to free text comments. The free text table is referenced by the specimen table to store specimen lab notes. The specimen test table references it for test notes. The component table references it to store component comments and test results that are reported as multiple lines of text. Finally, the isolate and sensitivity tables reference it for culture specific comments.

*POCT Transaction* (*POC TRN*): The POCT transaction table stores the history of POCT device uploads to the data warehouse. This table stores the *machine identifier*, *date and time,* *hospital1*, *location within the hospital*, and *a transaction identifier* for each POCT device uploaded. The information in this table is used to monitor the frequency of POCT device uploads. The upload frequency can be sorted by device identifier, hospital, or location within a hospital.

The transaction identifier is used in the GTS and HEM tables, connecting the glucose and hemoglobin data, respectively, to several other transactions. This data schema provides an efficient mechanism for identifying collections of POCT data that were downloaded within a period of time and/or from select locations.

POCT Data (GTS, HEM): The POCT data tables store the contents of the POCT devices. These tables contain patient identifiers, operator identifiers, QC results, and test results. The patient identifiers can be used to link patients to lab results by cross-referencing with the patient table.

### 4.2. Data Structures Described in Integrated Frameworks

#### 4.2.1. Horizontally Varying Dimensions

**Figure 6:**
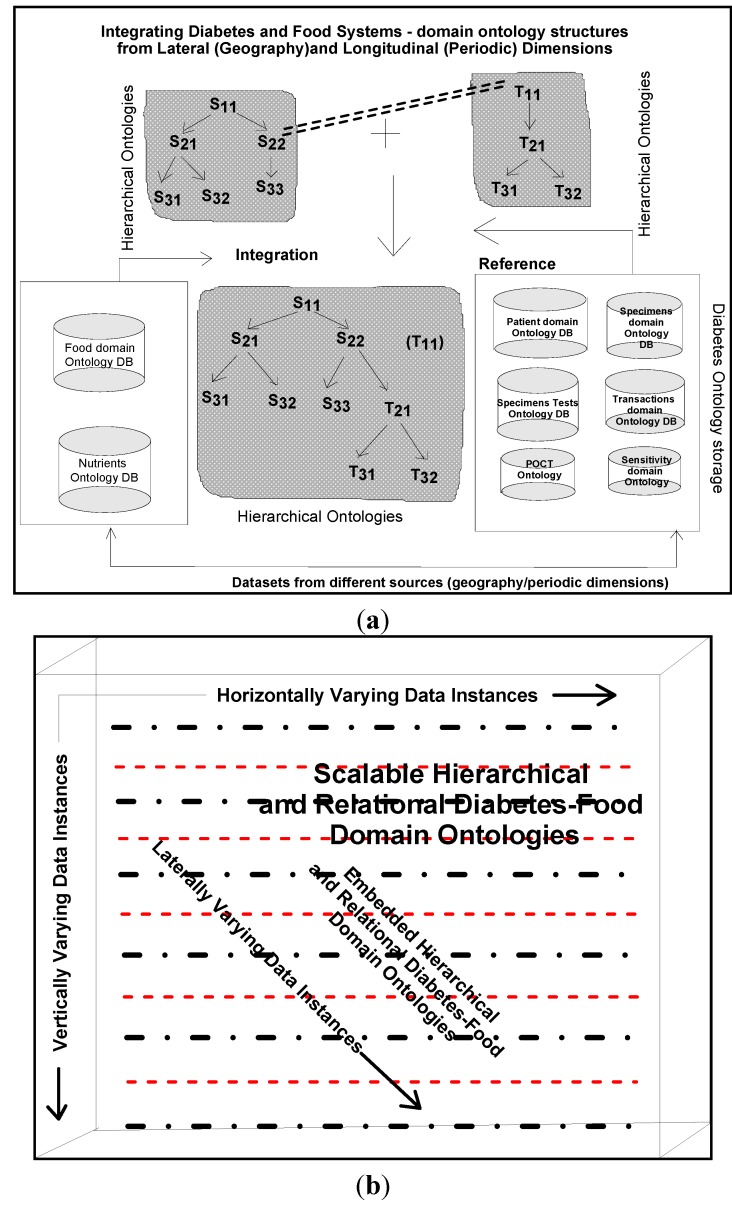
(**a**) A typical hierarchical data structuring model. (**b**) Horizontal, vertical and laterally varying data dimensions in 3D space *region*.

Each layer discussed in hierarchical data structuring, has distinct dimensions, related to a certain property. 3D region profiles (Figure 6a) describe *horizontal,* *vertical* and *lateral* dimensions. *Points*, *profile-lines* and *regions* are geometrical dimensions in a geographic domain, represented each in different hierarchies, as horizontally varying dimensions. 

Each *region,* which is inherently a composite dimension, consists of several *points* and *profile-lines*. The *point* dimension connects different domains of data, such as diabetic, food related and navigational data (that represent a geographic region or location). Similarly, *profile* and *region* dimensions are described. As shown in Figure 6b, horizontally and vertically varying hierarchies and their associated data instances are grouped from gathered data volumes. These grouped values are made interconnected through navigational data attributes, for which the coordinate data instances are described for all geometrical *point* dimensions of multiple *regions*.

#### 4.2.2. Laterally Varying Dimensions

In the case of laterally varying dimensions that are associated with lateral hierarchical structures, data layers are conceptualized and contextualized through laterally describing dimensions. For example, in diabetic data instances case, a structured geometrical *region* may have several data grids, from 2D and 3D populated line-profiles (geographically connecting populations locally and globally) that have multiple data in**s**tances grouped from laterally changing dimensions. As shown in Figure 6b, laterally varying dimensions are organized with their ontological descriptions. Thus, multidimensional data structuring procedures are followed up for horizontally and laterally grouping data grids and surfaces on all chosen *line-profiles* across geographic *region* dimensions.

#### 4.2.3. Vertically Varying Dimensions

As shown in Figure 6, data instances are collected from a profile that connects a geographic *region*, where several *points* are described in vertically varying dimensions. As described in Figure 6b, vertically varying dimensions are grouped on all profiles and thus computed grids and surfaces. These computed grids and surfaces across profiles represent vertically varying ontological descriptions. Each geometric dimension, either *point*, *profile* or *region* possess domain-data instances with hierarchically varying magnitudes in the vertical direction. When their instance values vary in this direction, the data structure changes into different relationship or as responses reported from conceptual domains, such as *dips*, *slopes* (*dip* and *steepness* are again dimensions) of the response curves measured from diabetic patients, as are discussed and interpreted in forthcoming Section 6, Section 7 and Section 8. 

In summary, mapping is carried out by arranging multiple geometrical dimensions with hierarchical structural representations, as illustrated in Figure 6a, normalizing and denormalizing [11] data relationships (evolved through conceptualizations, specifications and contextualizations). In this mapping process, diabetic clinical data, including results (data instances) are made connected through multiple domain ontologies in horizontally, laterally and vertically varying dimensions. In another procedure [12,11], such clinical tool measurements obtained, grouped across profiles (*line* and *region*-profiles) of patients (at both local and global perspectives) and their associated food domain ontologies are connected and integrated, as described and narrated in different schemas in the following sections.

### 4.3. Ontology Modeling

Ontologies are written to conceptualize and contextualize both existing (known data relationships) and emerging attributes (from unknown and or derived data relationships) of the dimensions and their classes that participate in the current modelling process. These are new descriptions, meant for interpretation and its refinement in each domain. Our work is initiated in the petroleum resources business domains and then extended in healthcare domains and applications. Ontologies written, are domain and application specific, and for connecting different *food-diabetes* domains, since these two compatible ontologies are integrable and mappable together, keeping in view that ontologies are shared in each other’s domain. 

Ontology is a formal representation of knowledge, based on specification of conceptualization and contextualization. Different entities, objects and dimensions and their relationships (either known or unknown) chosen, appear to be existing in the domain of knowledge representation and interpretation. Conceptualization is a simplified view of abstraction, but needs to be represented in knowledge domains, so that they can easily be integrated and shared by other related domains. Web ontology languages or even the existing archetype patters of high levels ontology descriptions [6], are used for describing semantics, schema, and syntaxes including system specifications and representations. They also address different naming conventions, vocabularies and contents including conflicts and ambiguities that arise during conceptualization of dimesions and structuring procedures and processes. Schemas and semantics of dimensions and their attributes can be swapped as long as they are cognitively associated domains, such as *food-diabetes* are associated dimensions. Our idea is to integrate different cognitive ontology specifications and representations. For this purpose, the concept of warehousing is used for integration, in which all the multidimensional schemas are compatibly accommodated, structured and integrated into a metadata.

*Golden software solutions* are initially used for drawing schemas and sub-schemas. Rules and constraints, templates, mappings, domains, formats, measurement types, instruments, dimensions, attributes, fields, treatments, patients, medical practitioners, medical practices, benchmarks, test results, specimens, specimen types, specimen sizes, periods and units are used in the ontology descriptions. Besides glucose levels, glucose type, ophthalmology and eye tests, daily food intake, types of food, food hygiene, normal daily routines, amount of time spent in daily exercises and cholesterol level measurements are incorporated in the modelling process. These multidimensional structure descriptions (ontologically conceptualized at places) are made connected and shared in the form of schemas narrated in Figure 3 and also in Figure 4 and Figure 5, as detailed in the following sections. 

### 4.4. Multidimensional Tools-domain used in the Diabetes Management

Glycemic Index Calculator, Diabetic Risk Assessment Calculator, HbA1c or A1c Calculator for Blood Glucose, blood sugar conversion, blood sugar chart, body mass index, waist to hip ratio, risk of heart attack, life stresses chart, Male sexual performance score. These are different apparatus dimensions, their facts and instances are used in the multidimensional data modeling and mapping process. 

All the relevant dimensions considered in the integration process of diabetes, cholesterol and food domain ontologies are analyzed and the known relationships among these ontologies are identified and how best relationships built are based on the hidden (not known and familiar) ontologies. How best, the ontology models described, are integrated to an standardized metadata, in a way usable for data mining purposes. Periodic or historical data are basic data for mining, identifications of existing rules among datasets and uncovering the unknown rules from the data including constraints. Unknown constraints and attributes are other interesting emerged dimensions in the data derived through conceptualization. Size of the data, number of dimensions evolved in the mapping and integration process, have definite impact on multidimensional data mining. Key attributes used in the multidimensional modeling of blood sugar levels are given, as shown in Figure 4. This is intended to be connected to the food-domain schema as narrated in the next section.

### 4.5. Description of Multidimensional Food-domain Ontologies

Food domain ontology (within the context of its associativity with diabetic domain) is aimed at understanding its connectivity with diabetes ontology and associated food domain ontologies, as suggested for diabetes meal plans, including the type, amount of nutrients and recommended daily food intakes. High insulin, carbohydrate food intakes and unhealthy life styles cause obesity, diabetes and their associated ailments. White bread, potatoes and white rice are high glycemic. Low glycemic foods such as whole grains, pasta, vegetables, fruits, beans, seeds, nuts and low fat cooking oils, such as olive and canola are chosen instead, for controlling weight, blood sugars and blood pressure. These foods possess much antioxidants, which can develop immune system and resistance to major illnesses. Americans, Europeans, Africans, Asians and people living in Pacific regions have different lifestyles and food intakes, based on their age. Based on these criteria, the real extent and magnitude of diabetic disease varies.

**Figure 7:**
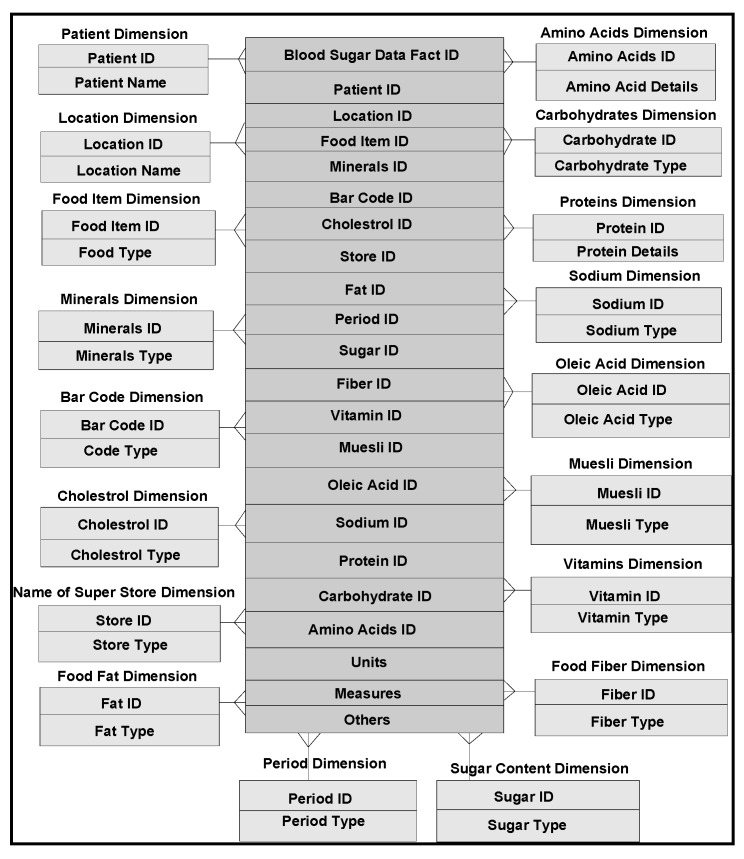
Multidimensional model of different qualities of foods that affect the blood sugar levels.

For example, though for sure the trend is changing, most American families do not prefer whole grains and vegetables on their shopping list. Europeans and Mediterranean people are vigilant on their diets, and especially prefer appetizing dishes made from vegetables, fruits and whole grains. Africans regularly consume medium to moderate fat meals with lot of vegetables, green bananas (steamed) and with lots of red meat including fish, fresh fruits and nuts. African populations, though cautious on sugar intake, are affected by high blood sugar levels. Asians, especially Indians include white sugar intake in their regular meals. Unlike Indians, intake of sugar by Chinese and Japanese is moderately low, especially among the aged population. Most of the sugar-related diseases are among Asian populations. As documented and interpreted in [13], there are 177 classes (dimensions), 53 properties (attributes) and 632 data instances, in food domain ontologies compatible for domain integration criteria. Countrywise data instances acquired worldwide, are populated within dimension and fact-data tables. As per schema design and business constraints narrated in Figure 7, instances from multiple dimensions and fact tables are mapped.

### 4.6. Domain Ontologies Representing Antioxidants

In addition to the awareness of healthy lifestyles*,* in recent years, *antioxidants* has become a new, exciting and rapidly expanding field of science. Information and knowledge on this growing science, need to be tied up with our daily routines. Certain foods such as *tomatoes*, *chocolate* and *green-tea* are healthful. What is being emphasized here, are the effects of the antioxidants found within these foods. Many of the chemicals that provide natural coloring to fruits and vegetables are antioxidants. The data instances relevant to anti-oxidants are populated within dimension and fact data tables, setting business rules, for each schema (Figure 5). There are literally several thousand of these compounds contained within foods. Their role in the prevention of cardiovascular disease, insulin resistance and perhaps even cancer, is poorly understood. Free radicals and antioxidants are commonly used in narrating disease mechanisms [14]. Free radicals are either derived from normal essential metabolic processes in the human body or from external sources. Free radicals can also form in the cells as a consequence of reactions between enzymatic and nonenzymatic consequences. In the case of diabetic patients, free radicals are produced from noxious chemicals (formed on blood vessel walls), generated from daily intake of food. Foods may have high glycemic or high cholesterol values, and free radicals can overwhelm blood vessels’ natural defences. A balance between free radicals and antioxidants is necessary for proper physiological function. If free radicals overwhelm the body’s ability to regulate them, a condition known as oxidative stress ensues. Free radicals thus adversely alter lipids, proteins, DNA and trigger a number of human diseases. This is why additional antioxidants absorbed during a meal can be so helpful. If fruits, veggies and whole grains are neglected in the diet, an extra protection for arteries is denied. Proanthocyanidins and ellagic acid in berries, indole-3-carbinol and β-carotene in broccoli, lycopene (of the carotenoid family) and glutathione in tomatoes, sulfur compounds in garlic, lutein in spinach, the catechins in green tea, and β-carotene (a member of the healing family of carotenoids) are effective in controlling sugar levels. Genistein and other isoflavones in soy and vitamin E, rich in phytic acid, known as IP-6, a potent antioxidant in whole grains are very significant antioxidants, not only effective for diabetic patients, but for cancer, cholesterol, heart disease, eye-related diseases and osteoporosis.

Mediterranean meal plans rich in vegetables, whole grains, fruits, nuts seeds, olive and canola oils, limited red meat and fish are low fat, while others are more moderate in their fat content. A moderate wine intake has been a feature of some studies [3]. 

### 4.7. Nutrition and Food-label Domain Ontologies

With today’s food labels in supermarkets, consumers get valuable guidance on food nutrients and required daily diets. Nutrition information about almost every food in the grocery store, with healthful food choices, is available by easy-to read formats. Information on amount per serving of fats, cholesterol, dietary fiber and other nutrients are major health concerns. Nutrient reference values with percentage daily intakes that can fit individual overall daily diets, uniformly describe food’s nutrients content, ensuring understandable relationships between nutrients, foods and diseases or health related conditions, such as diabetes and sugar, calcium, osteoporosis, fat and cancer. Standardized serving sizes making nutritional comparisons for similar products are helpful. Declaration of percentage of juice content in juice drinks, sugar content in juice, help consumers understand how much is required for daily intake and per serving. Food labels explaining uniformity of serving sizes of similar and dissimilar products for different healthy background people, similarities and comparisons among nutritional qualities of related foods are useful. Labels also provide grams of total carbohydrates, proteins, fat and sugar contents. Naturally present sugars in foods are healthful compared to manmade white sugars for managing diabetes. Labeling of grams of proteins intake is helpful for restricting protein intake either to reduce or avoid the risk of kidney disease. Label claims of low, saturated fat and high fiber are beneficial for diabetic patients. Other claims including diets low in saturated fat and cholesterol, rich in fruits, vegetables fiber-grains, and soluble fiber from whole oats, may help reduce the risk of coronary heart disease. These descriptions are incorporated in building schemas and sub-schemas, using all relevant multidimensional data instances. 

### 4.8. Integrated Frameworks

**Figure 8:**
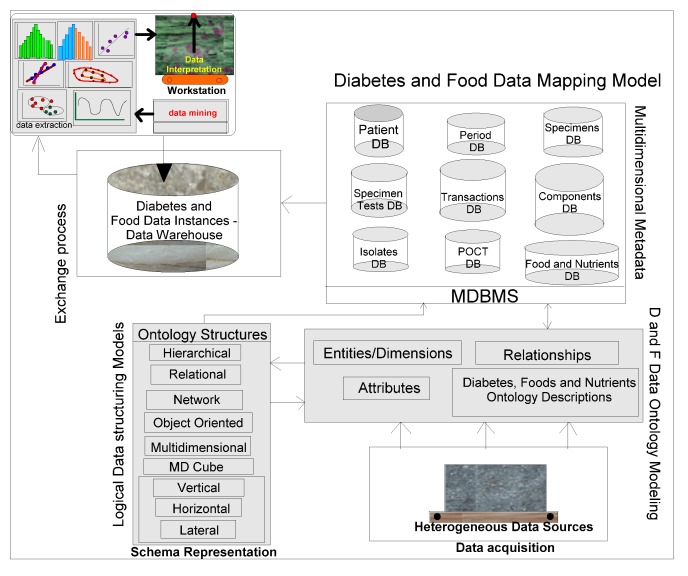
An integrated framework for connecting the diabetes and food ontologies.

*Grapher* (*Golden and Rockware software*) solutions are used to draw the multidimensional data schemas as represented in Figure 3, Figure 4 and Figure 5. Protégé 4, an ontology descriptive editor, describes multiple dimensions, fact tables, their attributes, their associations including population of data instances in dimension and fact tables. All the workflows, described in the paper, are initially designed by *golden software solutions*. Figure 8 depicts an integrated framework, starting from acquisition of data sources through conceptualization to implementation of conceptualized models through evaluation of models. It is a prerequisite that an ontologist, who describes the knowledge based ontologies, has an idea on domain knowledge and even evaluating models at intermediate stages of development. Cubes are normally generated either in Windows-based or Unix-based, Oracle driven workstations. 3D cubes are cartoons, drawn again using *grapher* editor and drafting utilities, to represent how multidimensional data is organized in the 3D data cubes. As demonstrated in an integrated workflow, all the sequence of events that occur from conceptualization to implementation are intelligently connected and stored. This framework works interactively on live data, in which description of data, data types, domains, structures, data integration (through data warehousing), data mining, visualization and interpretation, all are performed in a single canvas.

#### Robustness of Methodology

The discussed integrated framework and workflow have the ability to resist any changes in the selection of domains, the data types, number of dimensions, attributes and the data structures used in the modelling process. The authors demonstrate the feasibility of integrated workflows and their adaptability in any domain. These methodical approaches are initially applied in the petroleum business, human anatomy and human ecosystems, environment and disaster management domains and their versatility and flexibility evaluated [4,11]. Business rules and constraints used in describing ontology models in a particular domain, may have similar semantic, schematic, syntactic and even system heterogeneities, which are meant to be resolved by common and sharable ontological descriptions. Statistical procedures used for mining of correlations, patterns and trends among data sources offer consistent results for interpretation and evaluation of models. Archetype patterns and already existing high-level ontologies [6] can also be integrated in a warehouse environment that can fit within in our proposed integrated framework. 

The integrated framework is a system (Figure 8) that handles conceptual data modelling to its implementation through integration of heterogeneous and multidimensional schemas in multiple domains. Dimensions and their attributes are accommodated in multidimensional schemas, in which their corresponding data instances are organized and stored in an integrated warehouse environment (such as simple MS Access or Oracle 9i, a Unix-based database environment through an ETL process). Views extracted from mining procedures are used for visualization and interpretation. A methodical framework, as illustrated in Figure 8, addresses a rigor in acquisition of heterogeneous data sources, segregation and aggregation of attributes and their data instances of sugar level measurements including periodic food intakes. Multiple connections are explored and connectivity is achieved through the system hardware and software utilities. Though presently, the mining, visualization and interpretation are done independently, but intended to be developed a system, in which connectivity among all modules in the integrated framework is established. 

Multidimensional structures (as described in Figure 3, Figure 4 and Figure 5) are accommocated in this framework for integration and storage of data instances. Currently, the component result is used to store reference values for laboratory results and reports as blocks of text and microbiology culture results. Some laboratory results reported as multiple lines of text, are supported by creating a text identifier for the block of text, storing the identifier in the component result field, and finally storing the block of text in the free text table. Similarly, microbiology culture data are stored in a separate table. When culture results are reported, the transaction identifier stored in the component result column, creates isolates and stores in the isolate table with the transaction identifier. Sensitivity/MIC data instances are stored in the sensitivity table along with the isolate *id* instance. Using the transaction and component tables, the progression of microbiology culture results are tracked while maintaining a normalized schema.

Nutrient and health claims are only used under certain circumstances, such as type of food containing appropriate levels of stated nutrients. Similar multidimensional models are constructed connecting similar and or dissimilar *antioxidants, nutrient foods* and *food*-*labels* attribute cases which are not presented here, but used and represented in plot and map views for interpretation. Several issues associated with data mining, data visualization and data interpretation that lead to model implementation of drug prescription management are described in the following sections.

## 5. Data Mining and Knowledge Representation

Within data warehouse and mining environments, several events occur, for example, preventive care, diabetic medication, designing special meal plans, based on nutrients, serving sizes and antioxidant supplements. Mining rules and algorithms can compute several of such correlations, trends and patterns from historical data of diabetic ailments, with these preventive care events. Contextual ontology has more relevance especially building relationships among diabetes, foods and nutrients, with several contexts among these domains. Rule mining, decision tree structures, cluster mining, classifications uncover knowledge on medications and meal plans, from historical diabetic and foods domain data. In classification, association, sequential and spatio-temporal patterns are few examples. Classification patterns may provide description of the characteristics of the mass population, having certain diabetic related eye disease. They are then used to predict whether a new patient is likely to have similar symptoms. Similarity/dissimilarity and association patterns may indicate symptoms or treatments that often occur together, which can highlight on pictorial views, deduced from relationships with other domains.

**Figure 9:**
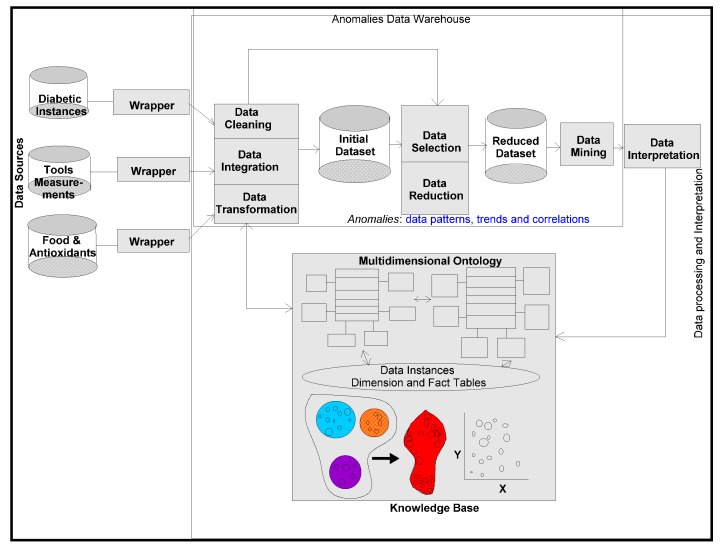
Knowledge management process model.

Uses of both geographic and demographic information presented to the doctors, are made to uncover impacts of age, race, region on diabetic disease. Doctors predict different meal plans based on age, race, region and the severity of the disease. Based on *comparison* and *similarity* ontology descriptions [11], doctors are able to tell which factor dominates in terms of the number of patients having a particular type of diabetes and eye disease and how many deviations on the ailments and symptoms can occur among the groups, over the number of years. Authors interpret the historical diabetes and foods domains data, the glucose levels affected by different types of food intakes. For this purpose, a knowledge management process model is designed, as narrated in Figure 9, to interpret data views and documented records. Glucometers that record the diabetes ailments of patients, are used besides physical observations of patients symptoms, such as frequency of urination, increased thirst and blurry vision. Pre-processing and post-processing steps can often be the most critical elements in determining the success of real-life data-mining applications. This fact is particularly evident in a recent medical application for diabetic patient screening. Many countries, about 10–30% of the population are diabetic. This disease has many side effects such as higher risk of eye disease, higher risk of kidney failure, and other complications. Patient information, clinical symptoms, eye-disease diagnosis and treatments, etc., are captured in a database. The availability of historical data leads naturally to the application of data mining techniques to discover interesting patterns. The objective is to find rules that can be used by medical doctors to improve their daily work; that is, to understand more about the diabetic disease and/or to find out how the disease might be associated with different segments of the population and its activities.

For knowledge representation and interpretation, identification of mining rules among causal and periodic data relationships, is considered. The doctors involved with the data mining projects [11] confirm many of the rules and causal relationships. They document data patterns and trends that were observed in their practices. Doctors were also surprised by many of the exceptions that they did not know before. As a result of data mining, the doctors gain good understanding of how diabetic disease progresses over time and how different treatments affect its progress. Special low-fat diet plans designed, based on these studies that control blood insulin, body weight, blood pressure and cardiovascular problems, have rapid effect on the health of a person and thus save millions of dollars among mass populations.

**Figure 10:**
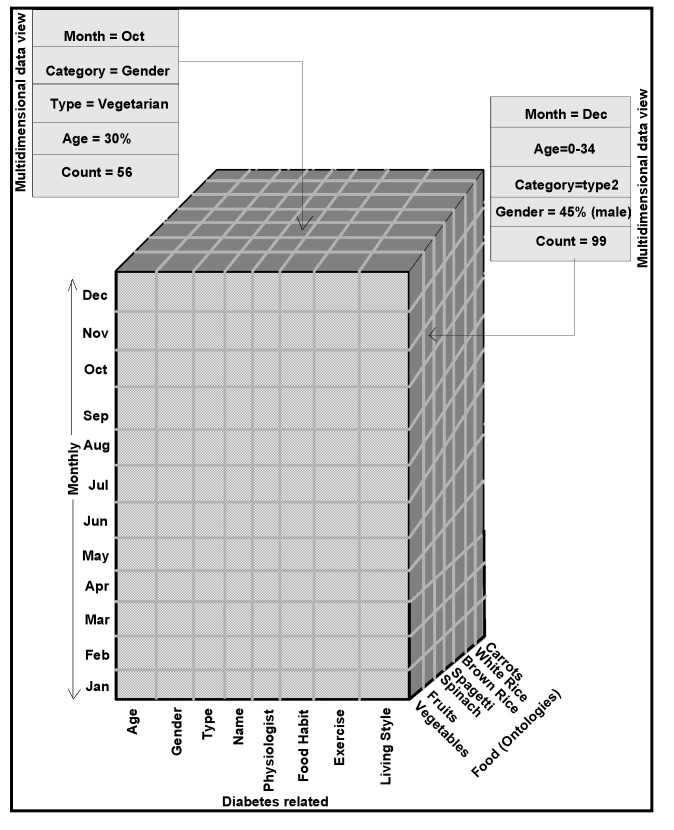
Data cube taken from an integrated warehouse metadata structure and extracted data views.

Rule mining, decision tree structures, cluster mining, classifications are different clues [11] for uncovering the data patterns, correlations and trends. Once the data are cleaned, the mining process begins. Many data mining techniques are now available [15] to discover patterns in the data sources. In general, the discovered patterns fall into one of the following types: classification, association, sequential, and spatial-temporal patterns. In the diabetic database application, we focus on the mining of classification and association patterns. Classification patterns provide a description of the characteristics of the population having certain diabetic related eye disease. They are then used to predict whether a new patient is likely to have an eye disease. Association patterns provide a list of symptoms or treatments that often occur together, which give a complete picture of the relationships in the domain. It might be desirable to detect patterns defined by events, such as insulin use, and not only by parameters, such as glucose levels. Such patterns might generate more meaningful interpretation contexts. Slices are extracted from cubes (volumes created from gridded surfaces, as explained in Section 4), as data/map views for visualization and interpretation, as demonstrated in Figure 10. 

Authors attempt to process, narrate and interpret the medical examination results in the form of traces. These traces are recorded by different instruments that continuously monitor the diabetic situations of the patients including insulin levels. Glucometer can record responses of patient. *Frequent urination*, *increased thirst* and *blurry vision* may be quick symptom data views of the diabetes. 

**Figure 11:**
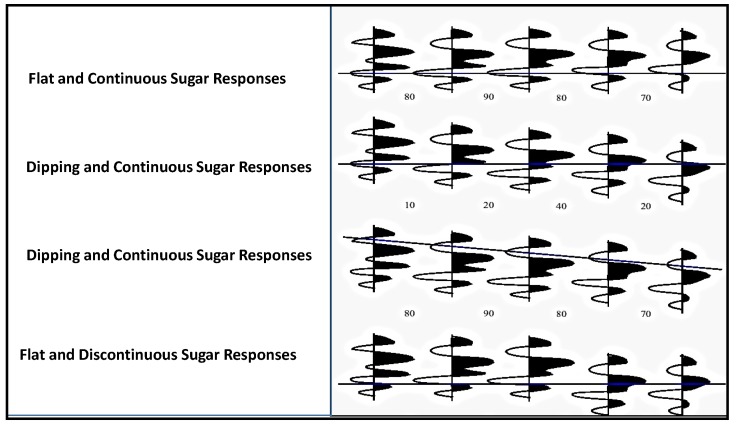
Analysis of sugar levels attributed by traces.

**Figure 12:**
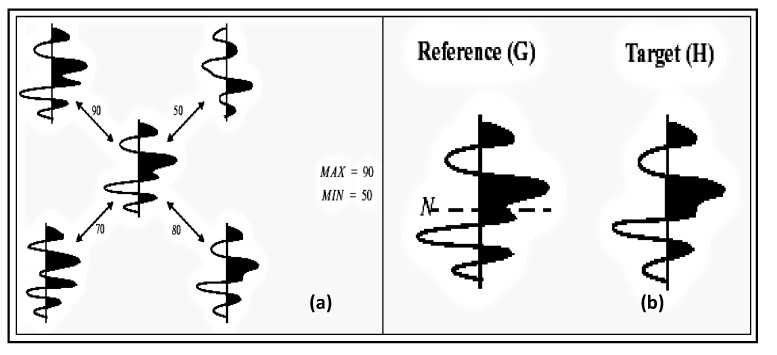
(**a**) Four-trace analysis patterns. (**b**) Performs Manhattan distance analysis for center trace to target trace, selected from the semblance scan process.

A state-of-art data mining tool that integrates classification with association rule mining (CBA) is used to find all such patterns in the data [4]. Authors use minimum support of 1% and minimum confidence of 50% as suggested by the doctors to mine association rules. Approximately 700 rules are generated in total. The doctors were totally overwhelmed. Some kind of post-processing is needed to help the doctors understand these rules. The mining methodology aims at giving doctors, a better understanding of the data and interpretation of discovered patterns. Thus mining models help the doctors to step through the massive amount of information in stages. For example, the analysis of samples or traces (as visualization is easier for interpretation), done for individual traces or traces of or records of different domains, is represented in the form of correlograms. As represented in Figure 11 and Figure 12, traces of samples or responses in the form of wavelets analyzed are patterns of flat, similarity, continuous and discontinues dipping events or traces. 

The *similarity* analysis in Event Similarity Prediction (ESP) [4,11] compares the center trace in a pattern of one to a maximum of eight surrounding traces (neighbors). Here trace is interpreted as response characteristics of blood sugar, blood pressure or any other response that narrates in the form of peaks and troughs of wavelet response. We choose the scan pattern and specify the length of the data to be included in the sliding comparison window. If events are dipping (Figure 11 and Figure 12), the program can search within a specified range for the best match. Semblance values are calculated for each reference trace to its neighbor trace pair within the scan pattern. From all these measurements, a single, target, neighbor trace is neglected. A second calculation computes Manhattan distance dissimilarity using the central trace and the target trace in the comparison window. This Manhattan distance value is output for the middle sample of the center trace window. ESP scales the Manhattan distance output. The dissimilarity of attributes may be computed from the Mahnhattan Distance between two traces “*G*” and “*H*” of samples:

*M_d_* = 100(Ʃ (*G_k_* - *H_k+d_*)/ (Ʃ (|*G_k_*| + |*H_k+d_*|)) (1)

Ʃvaries from *K* = *N* - *n*/2 to *K* = *N* + *n*/2 (2)

where *M*_d_ = Manhattan distance; *N* = the number of samples in the wavelet; *D* = the integer sample shift; *N* = the center sample of the reference trace; 100 = scalar to facilitate display.

Formula for calculating the Semblance Values between any two pairs of traces:

*S(t,d)* = 100(Ʃ (*G_k_* + *H_k+d_*)^2^) / (2 Ʃ [*G_k_*^2^ + *H_k+d_*^2^]) (3)

Ʃvaries from *k* = *t* - *N*/2 to *k* = *t* + *N*/2 (4)

*G* and *H* are two different data instances from two different wavelet responses.

Low *similarity* can result to steadiness in blood glucose levels. Steadiness includes either in high or low blood sugar levels. Contrasts in peaks and troughs among several wavelets plots suggest periodic changes of blood sugar levels. Contrasts in trace character are due to changes in glucose levels. Highly dipping events suggest a lack of trace coherency and similarity with poor data quality. A Manhattan distance dissimilarity value is computed using the center reference trace, the target neighbor trace, and the value output at the middle sample of the center trace window. These attributes are useful for detecting lateral trace changes caused by sugar level changes, including sugar related ailments. A dataset of Manhattan dissimilarity values has a range of 0 to 1. These values are then scaled by multiplying 100, to facilitate displays and visualization for interpretation. High values are areas of high dissimilarity and can indicate areas of rapid changes such as, blockades in the heart vessels. Average or median values give a view of the representative data similarity in the area. Low Manhattan distance values indicate a very uniform (low dissimilarity) sugar level. Low semblance or high Manhattan distance values do not always indicate sugar related blood vessel changes, but may have resulted from a low signal to noise ratio or poor data quality. 

Similarity is a sugar-level attribute that quantifies variance of the age, food habits, geographic location, together with a window under analysis. Similarity is like parallelism with the additional factor of sample spacing. Larger values imply greater similarity, smaller values, suggesting dissimilarity. Similar traces are characterized by relatively constant dip and azimuth as well as constant sample or response instance spacing. Dissimilar reflections are categorized by high variance in dip and azimuth or in glucose instance spacing, or in both. Similarity is used to understand glucose parallelism and continuity. Similar glucose instances indicate diabetic situations in a low energy environment, suggesting blockades. Dissimilar glucose levels indicate diabetics in a high-energy environment, which could suggest no block in red blood cells. 

An attribute quantifies how parallel sample instances are within a multidimensional window. Parallelism is a measure of the variance of the dips and azimuths from the average direction. The more uniform the dips and azimuths are, the greater parallelism and the more variance in the dips and azimuths, the less the parallelism. Highly parallel response patterns indicate diabetics in a low-energy environment, suggesting blockage of blood cells. Non-parallel responses indicate diabetics in a high-energy environment, which could suggest controlled sugar levels.

Waveform classifier clusters trace along a profile into a representative classes to produce a waveform class map. Waveform classifier is a response waveform classification solution. It works on the assumption that like waveform peak and trough instances correlate with diabetic data instance patterns. Using Manhattan Distance, wavelet responses are considered similar if their correlation coefficient is within the threshold of this window. Manhattan distance is an efficient statistical measurement of *similarity* or *dissimilarity*. Manhattan distance uses two equal length waveforms with N time samples and sums the absolute value of the difference in corresponding samples for all samples. Manhattan distance is given by:

*M* = Ʃ[*A_I_* - *B_I_*] (5)

where *I* = 1 to N; *M* = Manhattan distance; *A* and *B* are two data instances from different wavelet responses; *N* is the number of samples.

## 6. Data Visualization

### 6.1. Visual Analysis

**Figure 13:**
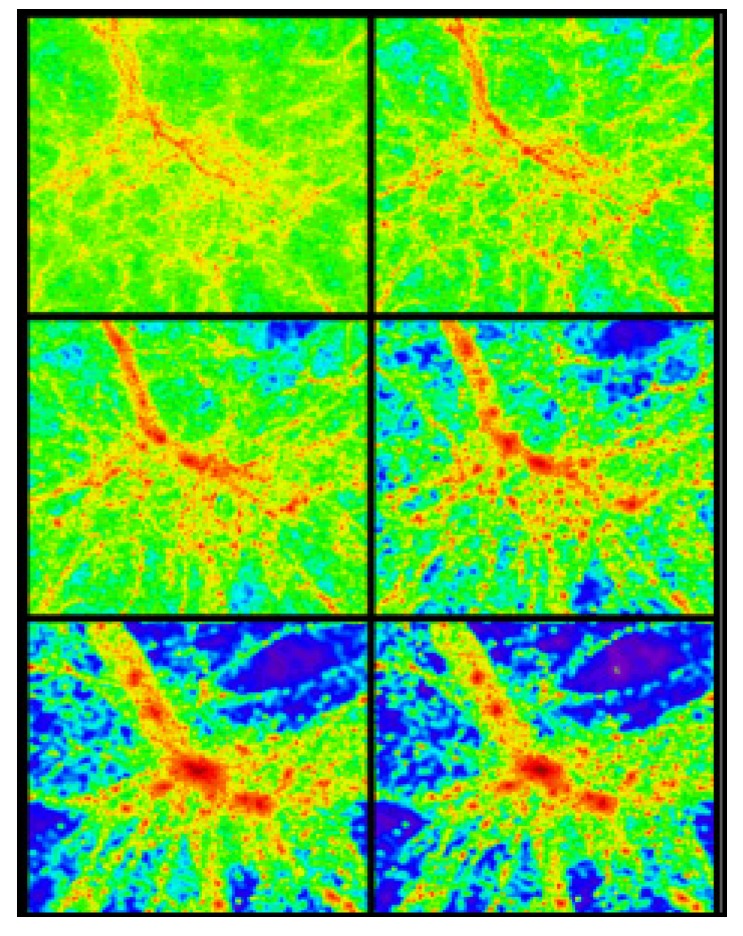
An example of diagnostic map view, showing red-blood density patterns.

Metadata that describe ontologically described multidimensional warehoused data support the data mining and visualization processes, which can chain the visual thinking. Visual thinking influences more on brain, with pictorial representation of processed or organized data, which is much better than representing in tables containing numbers. Grids, as narrated in Section 4.2, are used for computing surfaces. One of such surfaces, as shown in Figure 13, is an example, narrating thicker and thinner red blood density patterns, signifying glucose effects. The right presentation of logical information and its visualization make patterns simple for interpretation. Features, trends and outliers show up visually better way for interpretation, otherwise they never do with simple rows and columns. Simple pictorial map views depict the glucose data instance variations within a human body and with respect to periodic and space attribute dimensions, as described in the following sections.

Visualization of multiple domain data, such as sugar levels, food data instances and other associated data are represented in 2D and 3D map views in interpretation windows. Several profiles and their respective section views are displayed in 2D and 3D windows for analysis. Diabetic-food domain ontologies of *similar*, *dissimilar*, *comparative*, *non-comparative* data instances (as narrated in domain model in Figure 1) are aggregated based of *geography*, *gender*, *race* and *age* attributes. Integrated metadata are mined for understanding *anisotropy*, *heterogeneity* and *similarity* of properties of attributes from 2D plots, map views, variograms, correlograms, histograms and krigging. Krigging [10] is a special statistical procedure used with variograms, or two-point statistical functions that describe an increasing difference or decreasing correlation among sample values as separation between them increases, to determine the value of a point in a heterogeneous grid from nearby known values. In a multidimensional heterogeneous data analysis view point, a two-point statistical function describes an increasing difference or decreasing correlation, or continuity, between sample values, as separation between them increases. The term variogram is sometimes used incorrectly in place of semi-variogram. These two differ only in the semi-variogram that uses each pair of data elements only once, whereas the variogram uses all possible data pairs in other cases. Semi-variograms are typically used instead of variograms, but opposite vector directions (for example, north and south) are recognized as representing the same thing and having identical ranges, nugget points and the like. Similar analogy is made between *dip* and *flat* (profiles analyzed and taken from multiple domains) response curves with their attributes and properties, either may be increasing or decreasing in multiple directions, including similar multiple trends and correlations interpreted among other data attributes. These are graphical representations in which trends and correlations are brought out for *diabetic-food*, composited knowledge representation and interpretation. Handling large amount of data, providing consistent analysis in multiple dimensions, visualizing multiple diabetic-food data, integrated view interpretations and assessing uncertainty, are other purposes of visualization tools. Different visualization tools, used in the current studies also depend on characterization of metadata that is meant for interpretation and drug prescription management [4,5].

### 6.2. Interactive Data Exploration

Different patterns are interpreted during visualization analysis, to identify outliers. It is better to work with data views (directly shifting among different data views) without much support of more IT tools. A well-designed data visualization application supports analytical reasoning through easy-to-use interfaces. It allows to filter, rank, sort and group data; to calculate new fields from newly interpreted data patterns, correlations and trends.

### 6.3. Collaboration and Sharing

Analysis obtained from interpreted views (narrated from warehoused metadata), if unshared by different domain experts, makes clinical analysis futile. Analysis done by numbers is simple, but without any knowledge of the view, it may be hard to explain about the view. Good visualization provides story more naturally. Each and individual expertise becomes deeply involved, better narration of the story of the data view. Analysis that goes unshared or ignored is often as good as no analysis at all. That occurs too easily, when the story is told in numbers, making the story is hard to explain. Good visualization facilitates interpretation of records. Visualization of map or OLAP views [11] that depict the blood sugar levels of different age groups, different geographic regions, periods and different foods are easy to interpret, even for the clinical physiologist for prescription medications. 

### 6.4. Users of the Current System

The system developed is versatile and robust, so that doctors, patients, even social welfare and government organizations can use these systems. Even patients may be users, who can participate actively in documenting his/her records into the system that enables to interact with a domain specific medical practitioner in the local area. The system can accept limitless geographic and periodic data instances for any number of dimensions and their classes, to incorporate in the models. Especially, looking at the pandemic diabetic disease at geographic scale and population size worldwide, continuous and constant *doctor-patient* interaction can be made easier and versatile. Preventive care may be a prerequisite, since patient has more control on his/her own blood sugar and cholesterol levels, prior to start of his/her avoidable and expensive medications. Exhaustive documentation in a warehouse environment facilitates access to the patients’ hereditary histories including remedies. Even users of archetype patterns narrated in [6] can interact and incorporate their ontology descriptions using our robust integrated framework. 

## 7. Data Interpretation

In addition to data mining, data visualization and data interpretation, skill and expertise are significant for implementing and validating warehoused metadata. Patient specimen results and their interpretation are the responsibilities of medical practitioners, for medication and treatment of patients’ disease. Besides, appropriate medication as per the knowledge acquired from the warehoused metadata of diabetic data instances, a particular meal plan is triggered from food domain ontology. The environment in which patients have been living, is also an important criterion to be tracked within this domain and connectivity. Diabetes, cholesterol, food, and environment are different dimensions in domain ontologies that cannot be isolated since they coexist collectively in an ecosystem [4,10]. Secondary data collected from published sources and on the internet, are used for modelling and analyzing the mining data views. Different map views drawn from the data warehouse, are plotted and interpreted for knowledge extraction, as done in the following sections.

## 8. Results and Discussion

The framework is meant for connecting different modules, such as data acquisition, ontology descriptions, structuring, integration and metadata, data mining, visualization and interpretation, all performed together. It is an Oracle-driven package, in which some modules such as mining, visualization and interpretation, at present are independently performed (using grapher and surfer solutions) in the sense that more attention and skills are needed for visualization and interpretation of heterogeneous and multidimensional data types of spatio-temporal (both geographic and periodic dimensions) nature. Such types of data chosen in other business domains [8,9,10] are commonly, spatio-temporal multidimensional and heterogeneous in nature. *Similarity* attribute is interpreted in other domains, such as healthcare, environment and medical domains, in which not much work has been done using the current frameworks. The results obtained with different frameworks are intended to be compared, once framework development achieves its objectives, when connectivity is achieved among framework modules.

**Figure 14:**
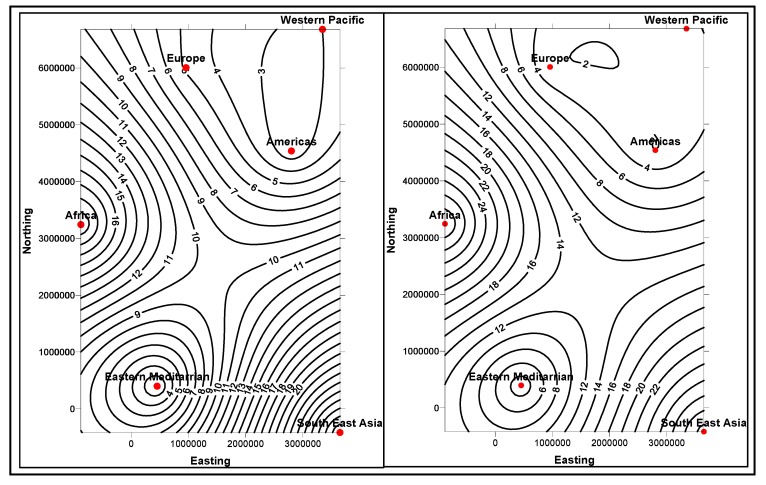
Map views for different regions showing diabetes deaths (0–34 age group of male and female, in thousands).

**Figure 15:**
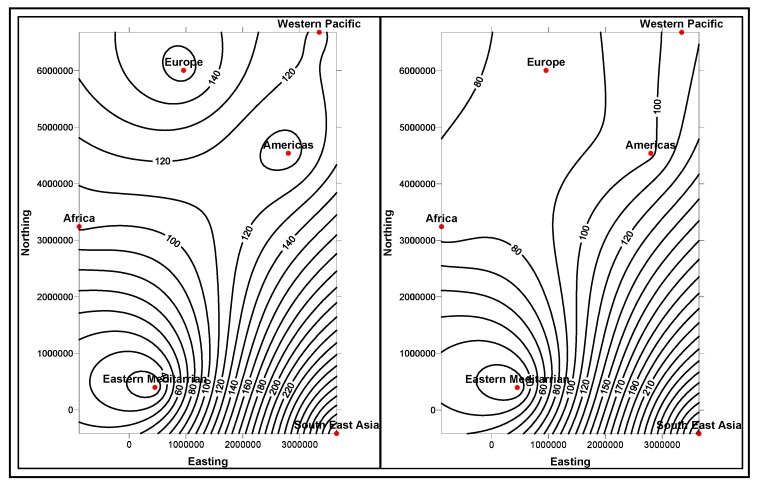
Map views for different regions showing diabetes deaths (34–65 age group of male and female, in thousands).

**Figure 16:**
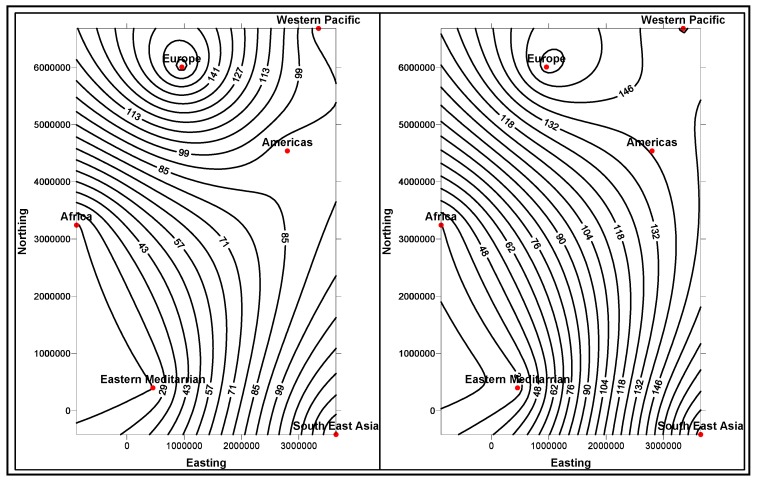
Map views for different regions showing diabetes deaths (more than 65 age group of male and female, in thousands).

**Figure 17:**
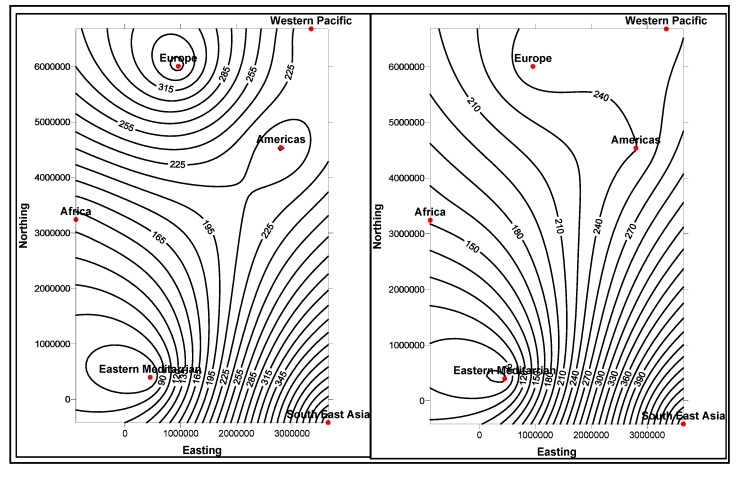
Map views showing diabetes deaths for different regions (male and female, in thousands).

**Figure 18:**
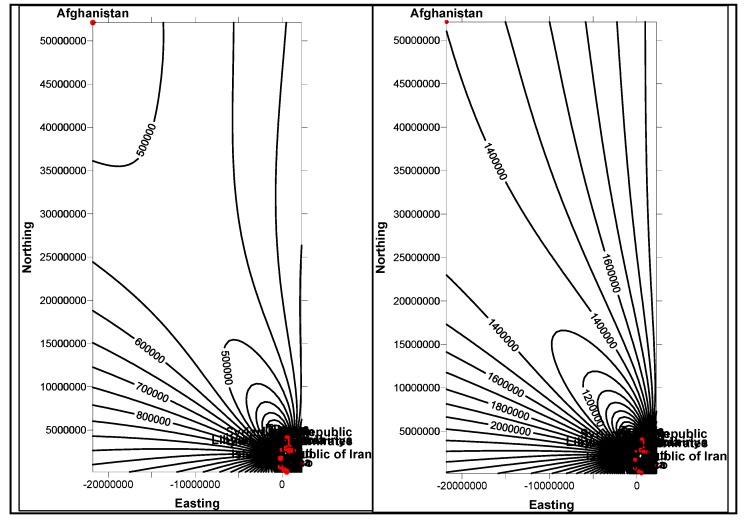
Map view showing diabetes reporting for Eastern Mediterranean region (for years 2000 and 2030 predictions).

**Figure 19:**
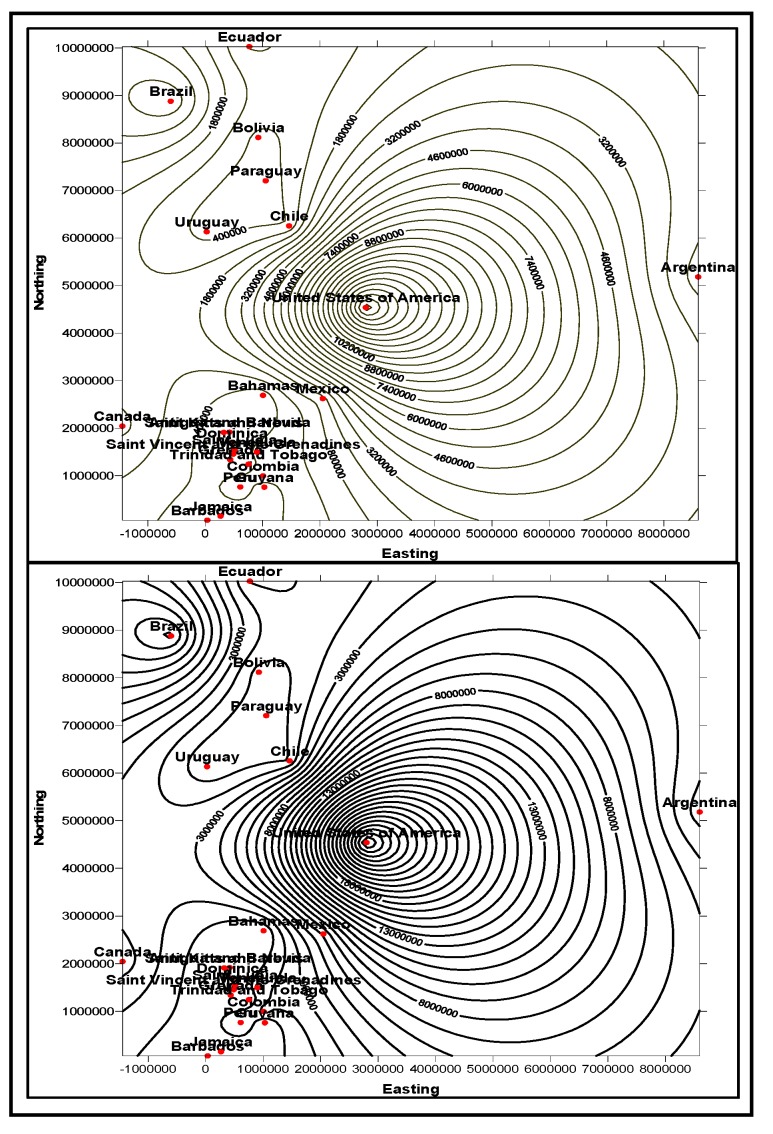
Map view showing diabetes reporting for Americas region for years 2000 and 2003.

**Figure 20:**
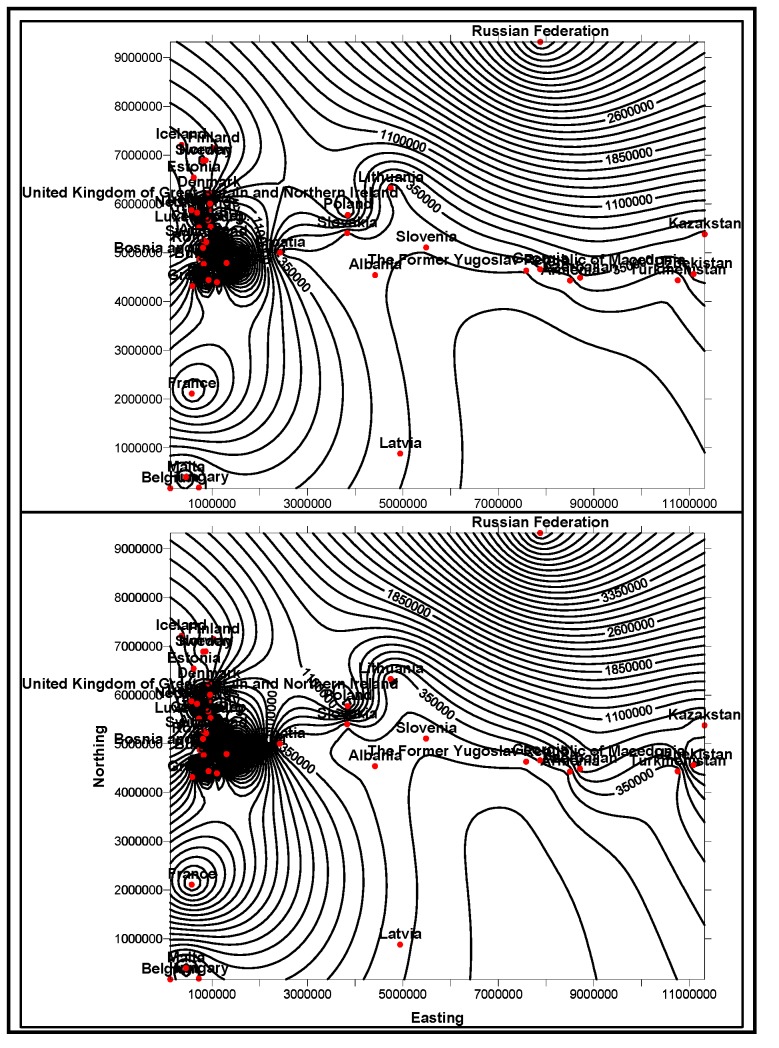
Map view showing diabetes reporting for Europe region for years 2000 and 2003.

**Figure 21:**
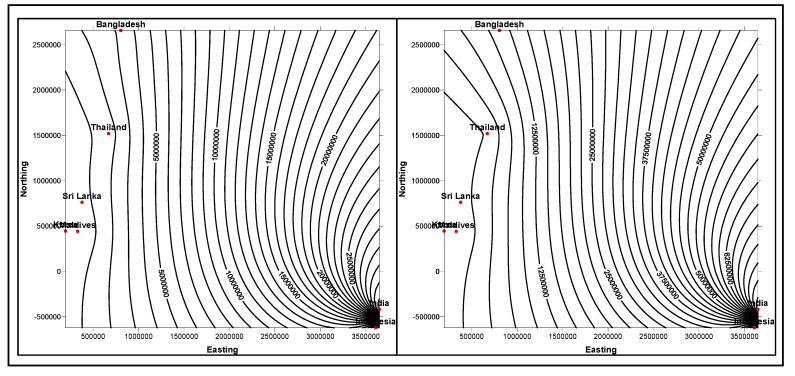
Map view showing diabetes reporting for SE Asia region (for years 2000 and 2030 predictions).

In the first stage, basic demographic and periodic information about the patients are presented to the doctor analysts. The goal here is to allow the doctors to know the basic information and understanding of the patient, how different attributes, such as age, race, etc. impact the disease and also the circumstances that population is living around the patient. The information is presented in several data mining views, for example, histogram graphs, and plot and 2D/3D maps views, for easy interpretation. Two types of information can be displayed: the absolute proportion or the relative proportion. Data instances collected worldwide, suggest as shown in Figure 14, Figure 15, Figure 16, Figure 17, Figure 18, Figure 19, Figure 20 and Figure 21, the proportion of the different race-age populations is displayed, having eye disease. The race-age group with the highest disease proportion is assigned to be 100%, while the rest of the group proportions are adjusted accordingly. Each bar represents an age group while different colors within a bar denote different races. This provides a common basis for comparison. In one glance, the doctor is able to tell which factor dominates in terms of the number of patients, having a particular eye disease and how much are the deviations among the groups over the years.

The map views computed and interpreted, are comprehensive as narrated in [11]. Data views (as represented in different map views, Figure 14, Figure 15, Figure 16, Figure 17, Figure 18, Figure 19, Figure 20 and Figure 21), extracted from warehoused metadata, are interpreted. The maps views, drawn from the *Grapher* solutions, have shown interesting data correlations and trends among diabetes and food-domain visualizations and their integrated interpretations that are described in different *geographic* regions and among different *age* groups. 

As an example, in areas geographically distributed, such as Africa, Asia, Latin America continents as shown in Figure 14, Figure 15, Figure 16, Figure 17, Figure 18, Figure 19, Figure 20 and Figure 21, people have similar lifestyles, with similar ailments and similar or dissimilar food habits. Similar age groups (such as 45–55 age groups) have similar diabetic type symptoms and or prescriptions. Adults have diabetes type 1, with similar age and food-habit groups or categories. These events are *frequency of occurrences* of diabetic symptoms over a *period of time* under analysis. Other events could be *blood-sugar* and -*pressure* levels under definitive age and gender groups. 

As per the map views shown in Figure 14, a diabetic death rise is observed for the 0–34 age group in African regions, and a decrease in their numbers is observed in The Americas, Europe and Eastern Mediterranean regions. Surprisingly, the number of females is more than that of males. As shown in Figure 15, on the contrary, the deaths reported among the 34–65 age groups is on rise, especially in the number of males compared with females. As demonstrated in Figure 16, an increase in diabetic-related deaths is observed in Europe among the age groups of 65 year or more, compared with the Eastern Mediterranean and Africa regions; this number is more among females. As reported in Figure 18, there are more diabetic cases reported in the Middle East compared with West Asia and prediction analysis towards 2030, in general suggests the number of diabetic cases is on rise in Asia, compared to Africa and Europe. Figure 19 demonstrates that in North America more diabetic cases were reported for both male and female genders during the 2000–2003 period, compared to Central and Latin America. Figure 20 indicates an increase in the number of diabetic cases (both male and female) in Russia compared to Europe in the same time period. A similar increase in diabetic cases is observed in the Asia-Pacific region (Figure 21). 

**Figure 22:**
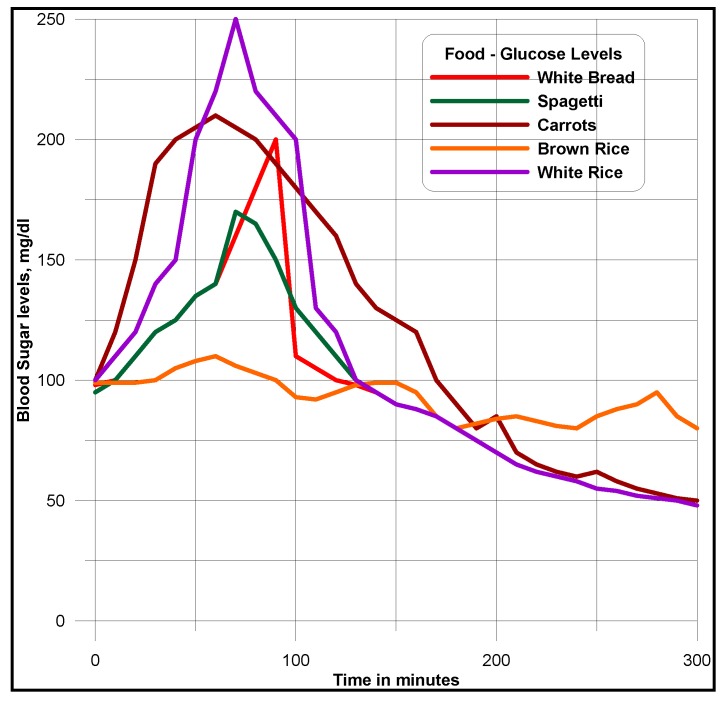
Blood glucose levels for different food types.

**Figure 23:**
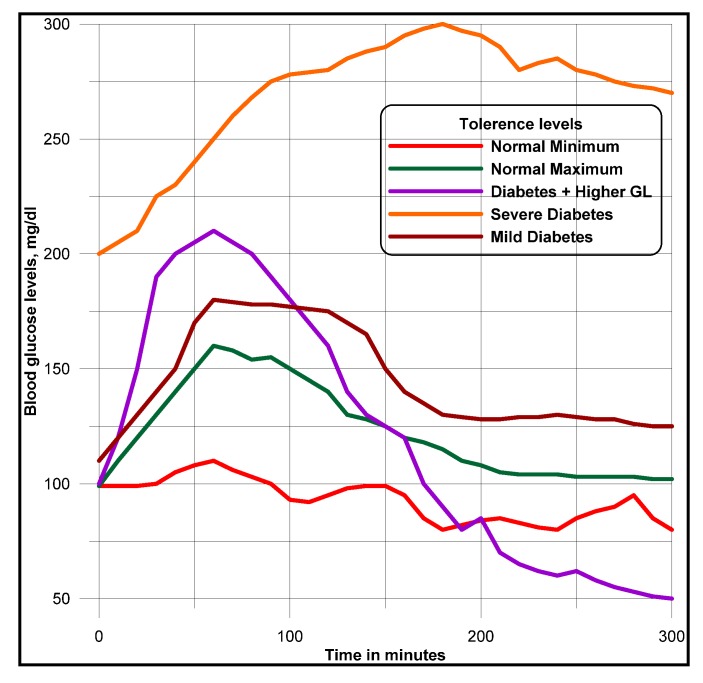
Blood glucose tolerance tests (5 h).

In the current reporting system, data instances are populated in several dimension and fact tables. Instances of attribute dimenions, such as *blood glucose levels,* are entered in dimension tables, along with *date, time of day’s* measurement instances in different columns of the fact table. A space column allows users to enter comments, such as medical practitioners’ *notes* on *changes in diet*, *daily routines that affect blood glucose levels*. As demonstrated in Figure 22 and Figure 23, high peak of glucose levels are indicated with white bread compared with lower glucose with *spaghetti*, which may even dip below normal with high-glycemic foods. 

The integration of different domain ontologies using the concept of warehousing has wider scope, keeping in mind the nature of multidimensional and heterogeneous datasets worldwide. Existing ontologies can also be used integrating in the current warehouse framework, investigating it using web ontology languages (OWL).

## 9. Conclusions and Recommendations

Though the present work is in progress and inconclusive, the Authors publish data warehousing and mining procedures compatible with visualization that narrates various data and map views, for interpretation and thus designing preventive health care programs. Countrywise seriousness of the disease suggests a huge scope of health-care system development on a global scale and analysis. Medication plans may be accelerated for the advanced cases of diabetic patients and appropriate meal plans suitable for healthy life styles recommended. Ontologically described multidimensional data-warehouse and mining facilitate the data interpretation and knowledge extraction. This is a robust methodology for documenting and organizing the diabetic cases, food, nutrients and antioxidant ontologies and their integration, for mining documents and reports. Data mining and novel interpretation ideas facilitate the medical professionals, involved in various government diabetic related projects or schemes. Interpretation of map and data views enables understanding of historical diabetic medication, recommended foods and nutrients for diabetic patients on different meal plans. The connectivity among different domain ontologies is possible through multidimensional data warehousing approach. Serving and food labeling are effective means of communication, facilitating choice of food for diabetic patients. Today, diabetic experts, no longer recommend a single meal plan for all people, having diabetic disease. Instead, using these studies, flexible meal plans can be recommended taking into account a person’s lifestyle, diagnostics analysis and particular health needs. Medical practitioners, welfare institutions, social workers, nutrition specialists can effectively use these methodologies for e-health-care management systems. Existing domain ontologies can also be used in our integrated framework. Similar robust methodologies and their implementations may be generalized for managing other ailments and diseases, worldwide.
